# Infants’ transition from milk to solid foods - the lived experiences of first-time parents

**DOI:** 10.1080/17482631.2019.1693483

**Published:** 2019-11-20

**Authors:** Annelise Norlyk, Jette Schilling Larsen, Hanne Kronborg

**Affiliations:** a Research Unit for Nursing and Health Care, Department of Public Health, Aarhus University, Aarhus C, Denmark; b Section for Nursing, Department of Public Health, Aarhus University, Aarhus C, Denmark

**Keywords:** Infant food, feeding behaviour, parent-child relations, transition phase, descriptive phenomenological study, qualitative study

## Abstract

**Purpose**: During the transition from ingesting milk to ingesting solid food, infants substantiate their eating habits. The present study focuses on this transition. Specifically, it aimed to explore first-time parents’ lived experiences of their infants’ transition from milk to solid foods.

**Method**: The study is based on the descriptive phenomenological approach Reflective Lifeworld Research (RLR). Ten mothers and ten fathers were interviewed twice; when the infants were aged four to five months and again at seven to eight months of age. Data were analysed according to RLR principles.

**Results**: The findings show that the transition from milk to solid food is a demanding in-between phase. The physically intimate feeding situation is replaced by unfamiliar situations in which parents and infant are physically separated and new types of food are introduced. The process of feeding requires parents’ full attention and sensitivity towards the infant’s reactions.

**Conclusion**: The study highlights how shared parental experiences were reflected in frames for how a meal should normally proceed, including parents’ desire to create healthy eating habits and uphold harmony duringfamily meals

We suggest for health professionals to present parents with a wider frame of normality, especially as concerns the concept of what constitutes “normal” eating patterns.

## Introduction

At the age of four to nine months, infants should learn to eat food of different texture, taste and appearance (WHO, 2005, 2018). Current transitioning guidance from the World Health Organization (WHO) recommends initiating complementary foods of different textures, tastes and appearances in addition to breast milk at six months of age (WHO, 2005, 2018).

During the transition from milk to solid foods, the infant undergoes important processes of change during which eating habits are substantiated (Birch & Fisher, 1998; Brown & Lee, 2015). The course of the infant’s introduction to food other than milk may influence the infant’s development and well-being in the short and also in the long term (Blissett, 2011; Brown & Lee, 2015; Gunnarsdottir, Schack-Nielsen, & Michaelsen, 2009). Studies have shown a relations between the introduction of solid foods and the development of refusal to eat (Schmid, Schreier, Meyer, & Wolke, 2011), picky eating (Shim, Kim, & Mathai, 2011) and obesity later in life (Schack-Nielsen, Sorensen, Mortensen, & Michaelsen, 2010; Seach, Dharmage, Lowe, & Dixon, 2010). Consequently, the establishment of healthy feeding practices early in life is important to promote lifelong healthy eating patterns and parents play a significant role during the transition phase.

Research has stressed, however, that many parents struggle to identify the optimal approach for transitioning from milk feeds to solid foods, particularly first-time parents (Harrison, Brodribb, & Hepworth, 2017). According to existing research, 25–35% of new parents in Western societies experience eating-related problems in relation to the introduction of solid foods (Ammitzbøll, Thygesen, Holstein, Andersen, & Skovgaard, 2018; Bryant-Waugh, Markham, Kreipe, & Walsh, 2010). Early problems may affect the parents negatively and cause frustration and, potentially, also an increased tendency towards depression (Lucarelli, 2013; Rask, Ørnbøl, Olsen, Fink, & Skovgaard, 2013). Conversely, given that parents can influence their infant in the transition phase, early intervention may prevent early eating problems (Blissett, 2011; Nicklaus, 2009; Scaglioni, Salvioni, & Galimberti, 2008), thereby affecting the infant’s self-regulation and development with respect to the introduction of solid foods (Scaglioni et al., 2008).

The transition phase has been investigated thoroughly to determine the kinds of solid foods to which parents should introduce their infants in terms of nutritional value, taste and texture (Schwartz, Scholtens, Lalanne, Weenen, & Nicklaus, 2011). Further, both international and national reports have described how and when parents should introduce the transition phase from milk to solid foods to guide parents and ensure optimal transition (The Danish Health Authority, 2019: United Nations, 2002; WHO, 2005, 2018). However, research has established that these recommendations may not always correspond with the reality experienced by parents (van Dijk, Hunnius, & van Geert, 2009, 2012; Vereijken, Weenen, & Hetherington, 2011). Several reasons may contribute to explaining parents’ varying experienced degrees of success. Infants may progress differently and react in various manners from one meal to the next, and success depends on the interaction between the infant and its parents (Kochanska & Aksan, 2004; van Dijk et al., 2009, 2012). Furthermore, it can be difficult for new parents to handle the transition because different sources provide different information about infant nutrition, which may be perceived as conflicting messages by insecure parents (Harrison et al., 2017).

The literature review above provides important insights of the transition from milk to solid foods and shows that the transition is a critical event particularly for first-time parents. However, qualitative studies are lacking as concerns the complexity of the transition as experienced by first-time parents. This knowledge is of importance for health professionals as it can provide an increased understanding of the personal aspects related to the transition from milk to solid foods. Consequently, there is a need to explore the meaning of the transition on first-time parents’ lifeworlds. Hence, the aim of this study was to explore first-time parents’ lived experiences of their infants’ transition from milk to solid foods.

## Method

The present study draws on the descriptive phenomenological approach Reflective Lifeworld Research (RLR), which builds on epistemological assumptions from continental lifeworld theory in general and the philosophy of Husserl and Merleau-Ponty in particular (Dahlberg, Dahlberg, & Nyström, 2008). RLR aims at describing a phenomenon as it is lived, which in this study is the transition phase as experienced by first-time parents. The question of lifeworld meanings is the focus to form a general structure of meaning which *encompasses both essential meanings of a phenomenon and the individual experiences* (Dahlberg, 2019, p. 56).

The researcher adopts an attitude of openness to allow the phenomenon to show itself and approaches participants with an open mind (Dahlberg et al., 2008). Accordingly, the authors worked by way of a bridled process of understanding, which included a reflective process of going beyond our “natural attitude” of taken-for-granted understanding. In other words the intention was to slow down the process of understanding by retaining a sensitive and open mind in order to see the world differently rather than imposing theoretical explanations on the material (Dahlberg et al., 2008). Hence, prior to any application of theory, the RLR approach allows us to capture first-time parents’ lived experiences of their infants’ transition from milk to solid foods.

### Participants

Ten couples participated in the study, i.e., ten mothers and ten fathers. They were recruited from a Danish municipality and invited to participate by their health visitor. Subsequently, JSL, a former health visitor, contacted parents who had accepted to participate, in order to make an appointment for an interview. Inclusion criteria were first-time mothers and fathers who were living together and were Danish native speakers. As sampling within a phenomenological approach is concerned with understanding a phenomenon more deeply through adequate exposure to the qualities of the phenomenon that are described by those experiencing it (Dahlberg et al., 2008; Giorgi & Giorgi, 2003), the sampling strategy is about variations of the experience rather than demographic variation and size (Giorgi & Giorgi, 2003; Norlyk & Harder, 2010). The mothers were 25–40 years of age, the fathers between 24 and 50 years. The parents were interviewed twice. At the first interviews, eight couples had started introducing solid foods to their infant, but had limited experience in doing so, while two had not yet started. At the second interviews, all participants had experiences with the transition to solid foods.

### Interviews

To obtain expressions of the parents’ shared and potentially different experiences of the transition phase data were collected by joint interviews with both parents. Norlyk, Haahr, and Hall (2016) argue that joint interviewing with couples is particularly relevant when the phenomenon under study is a shared experience, as joint interviews can both deepen and broaden the content of the data collected. All interviews were conducted in the parents’ homes by JSL. The interviews had a duration of 45–120 minutes and were conducted in 2016. In the first interview, the infants were aged four to five months; in the second interview seven to eight months.

In accordance with the phenomenological approach, interview questions were prepared, but not predefined. The interviews were meanings-directed and characterized by a dialogue (Dahlberg et al., 2008) in which the parents were encouraged to use their own words and to talk about what really mattered to them during the transition from milk to solid foods. Accordingly, the interviewer approached the participants with an open mind taking nothing for granted (Dahlberg et al., 2008) to capture the first time mothers’ and fathers’ lived experience of the transition phase as detailed as possible. The first interview focused on parents’ experiences of the transition so far, if any. The second interviews addressed specific descriptions of parents’ lived experiences of the transition. The opening question was, “Please tell me about your experiences of your infant’s transition from milk to solid foods?” Additional questions were asked to deepen the mothers’ and fathers’ descriptions, e.g., ‘Please give another example of this’. The interviews were audio recorded and transcribed verbatim by the interviewer.

### Data analysis

Interviews were analysed in accordance with the descriptive approach of RLR. The analysis focused on discovering patterns of meanings and variations in order to describe a general structure of the transition from milk to solid foods as experienced by first time parents. In other words a description of the phenomenon’s essence and constituents (Dahlberg et al., 2008). The essence reflects the essentials of the phenomenon while the constituents elaborates on various nuances present in the original data and give the contextual flavour to the essence (Dahlberg et al., 2008). Thus, the essence reflects a higher level of abstraction than the constituents which reflect the particular meanings. Accordingly, the aim of the analysis was to describe a general structure of meaning that encompassed both the essential meanings of the transition phase and the participants’ individual experiences.

The analysis consisted of a three phase process involving whole-parts-new whole. The first phase refers to getting an overall sense of the data, while the other phases represent the deeper analysis (Dahlberg et al., 2008). Firstly, all the interview transcriptions from the first and the second interviews were read repeatedly to get a sense of the data as a whole. Secondly the transcripts were divided into meaning units. Every meaning unit was carefully reflected on to explicate all meanings that contained aspects of the transition from milk to solid foods. This search for meanings was characterized by an active and intensive dialogue with the text by asking questions such as “What is being said?”, “How is it said?”, “What is the meaning?” Furthermore, the dialogue was supplemented by critical and reflective questions such as “Is this the actual meaning or is there a different meaning?” As stated by Dahlberg et al. (2008), this open and critical attitude aims at achieving scientific objectivity during the research process.

Subsequently, the authors reflected upon variations in meanings, differences and similarities in the meaning units. Meanings that seemed to be linked were clustered into a temporary pattern to get a preliminary overview of essential meanings and their interrelated structure. This was followed by a process of critical reflection to synthesize the clustered meaning units into a new whole that clarified the essence and the constituents of the transition as experienced by the participants.

Quotes from the interviews are provided as examples of explicated meanings.

## Ethical considerations

The study followed the basic principles for research given in the Helsinki Declaration (World Medical Association 2013) and by the Northern Nurses’ Federation (2003). Thus, the participants received verbal and written information about the purpose of the study, their right to withdraw, anonymity and the confidentiality of any data given. Also, a written informed consent was obtained prior to all interviews. According to Danish law, formal ethical approval of the study was not required as the study did not contain biomedical material. The study was approved by the Danish Data Protection Agency (jr. no. 2015-41-3953).

## Findings

The essential meaning of the transition from milk to solid foods is characterized by being a demanding and challenging in-between process. The meal of the infant changes its familiar pattern. The physically intimate feeding situation covering all the infant’s nutritional needs is replaced by unfamiliar situations in which parents and infant are physically separated and new types of food are introduced.

The transition from milk to solid foods carries with it a high degree of responsibility for the infant’s new nutritional needs and intake. Cooking the infant’s food and feeding the infant in new ways demand both knowledge, time and effort. The feeding process require parents’ full attention and sensitivity towards the infant’s reactions to act and cooperate in harmony with the infant. This interaction requires an ability to balance creativity and control during mealtimes, especially as parents wish to give the infant a positive experience of food and meals.

The essential meaning is further elaborated in the five constituents described below: Striving to be a responsible and perfect parent; Achieving and missing information; Being in a state of alertness to read the infant’s reaction to solid foods; Generating idealized images as a frame of reference for “a normal meal”; Communicating meal-related values to the infant.

## Striving to be a responsible and perfect parent

In the transition phase the mother was implicitly considered as the main parent responsible for the infant’s food intake and meals. Further, being the main parent responsible appeared as a fundamental task that any mother was expected to manage successfully. Striving to be a responsible and perfect parent thus meant that the mothers, in particular, placed great demands on themselves to do their utmost to manage the infant’s meals and food intake successfully. This responsibility carried with it an obligation to do the “right” thing in the “right” manner; meaning that mothers, in particular, were concerned with all the details related to the infant’s food intake.

While the mothers appeared as the person in charge in the transition phase, the fathers seemed to take the role of the supportive sparring partner; e.g., by acknowledging her efforts.

Father: P [mother’s’ name] is so clever at varying his diet. She is awesome.

Mother: Yet I keep worrying if his diet is sufficiently varied … him being so small and eating such tiny portions …

Striving to be a responsible and perfect parent could create feelings of insecurity and concerns, e.g., that a responsible parent needs to cook the infant’s food from scratch and, for some parents, preferably using organic ingredients. Hence, to be a responsible parent meant to meet personal ideals about serving the infant healthy, varied and inspiring meals. However, as the meals proceeded on the infant’s terms, and if the infant preferred ready-made food, this might legitimize compromise on their ideals.

Father: You think you are a poor mother if you buy his food ready-made?

Mother: Well, I think it’s sort of cutting corners really, and going for the easy option to buy ready-made baby food … on the other hand, I kind of think “What the heck”, if that’s all the boy wants to eat in the morning, let him!

Being a responsible and perfect parent was also related to the infant’s responses to the food. E.g., if the child ate sparingly, this could cause the mother to feel that she was unable to manage her ultimate task, i.e., to feed her infant. Failing to succeed, triggered feelings of self-doubt, uncertainty and lack of control. This could make the meals so burdening that it affected the mother bodily; meaning that the mother’s well-being was closely related to the infant’s food intake.

Mother: For the past week or so, feeding him has made my stomach ache … I was relieved when I’d fed him his evening meal and he just needed a bottle to sleep on … it was a relief that there would be no more food-related dramas that day (cries).

## Achieving and missing information

During the transition phase access to reliable information on what to feed the infant was important for parents. However, having access to a large body of information without being able to judge its validity created a paradox of achieving and missing information.

When seeking information on food for infants in the transition phase both formal and informal sources were regarded as sources of reliable information by parents. Accordingly, both information from the health visitor, family members, friends and Facebook appeared as reliable information. At times, this information contained conflicting advice resulting in parents finding it particularly difficult to navigate in this information overload.

The paradox of achieving and missing information meant that parents could feel insecure as to whether or not they lived up to the information given about the “right” food at the “right” time. The mothers, in particular, being the main responsible for the transition to solid food, could feel uneasy and vulnerable by the experience of information overload and the potential problems regarding the validity of the information.

Mother: My friends link to a lot of articles on Facebook; that stresses me. Today, e.g., somebody linked to a text which I took to mean that you’re a bad mother if you feed your baby on anything else than mother’s milk for the first six months … With all the stuff you read, it’s unlikely that you can ever do things right. That’s why I think you can’t go completely wrong if you follow the guidelines to a fair degree.

Parents could have difficulties in assessing whether or not the infant was ready to be introduced to this or that particular type of food. Information and guidelines from authorities concerning food in the transition phase were perceived in a different manner depending on context. Parents struggling to get their infants to eat tended to stick to the information and guidelines offered by authorities, e.g., the Danish Health Authorities. Parents who perceived their infants’ intake to be sufficient tended to have a more pragmatic attitude towards guidelines and preferred to rely on their intuition. Further, common sense could overrule advice given by authorities; e.g., one couple wanted to familiarize their twins with the taste of cow’s milk although they knew that this was not officially recommended.

Mother: I give them water during the day and then they get half a glass of cow’s milk with their evening meal so they learn to recognize the taste, although we know it’s not recommended.

## Being in a state of alertness to read the infant’s reaction to solid foods

In the transition phase the infant’s responses to being introduced to solid foods at times differed from one meal to the next. Thus, every meal evolved differently and the meal situation could change over time, meaning that the transition phase was not linear. This challenged parents’ ability to read the infant’s reactions during the meal and to respond accordingly. When introducing the infant to new food parents were extremely worried that their infant might choke. Hence, food of a more solid texture was perceived as being potentially dangerous for the infant. It took courage to introduce certain types of food and parents used their intuition or tried to find relevant information.

During meals parents were in a state of alertness to read the infant’s reactions ready to take action. They balanced between taking action to save the infant and having the patience needed to read the infant’s reaction and wait and see if the infant was capable of handling the situation independently. By demonstrating to parents how to take action, the health visitor could give parents the courage needed to introduce food of a solid texture.

Mother: Two weeks ago, the health visitor stressed that he needed some real food now because he was still having mashed potatoes with meatballs. Well, yeees, I said, I know somebody who lost their daughter because food got lodged in her throat, so I worry a lot.

Father: But then the health visitor said: “Let me show you what to do. If something gets lodged in his throat and he cannot fix it himself, you turn him upside down and do this”.

Difficulties in reading the infant’s reaction could create a series of dilemmas, e.g., how to relate to the risk of overeating versus under-eating and whether or not the infant should be offered extra food, a bottle of milk, etc. This created a sense of insecurity and vulnerability, especially among the mothers who seemed to internalize her lack of success which, in turn, contributed to her fear of not meeting the expectations of being a good mother. Further, being unable to read the infant’s cues could cause disagreement between parents.

Mother: It can be hard to know if he gets enough food; sometimes he wants only one bowl of porridge, other times he wants two … sometimes he whimpers and I feel really insecure. What if he is still hungry? THEN I’d be a terrible mother if I haven’t given him enough to eat. (…) We sometimes disagree on that. G (father) sometimes says “relax for heaven’s sake, he has had plenty to eat”, but I think that babies cry when they are trying to tell us something.

## Generating idealized images as a frame of reference for a “normal” meal

For parents the transition phase carried with it a high degree of responsibility for the infant’s nutritional needs and intake. No matter how easy or difficult the patients found that the transition phase was, they worried about their infant’s intake of food and vitamins, the quality of the food, etc. Personal and idealized images appeared as a frame of reference for a “normal” meal that parents relied on. Parents generated this idealized image from guidelines, movies, family, networks, etc. The meaning of this frame for “normal” constituted a script, including the size of meals, the infant’s interest in the food and how meals should proceed. If this image of “normality” deviated from the parents’ actual experiences with their own infant, they became insecure. Furthermore, if the parents’ images differed from each other, this could be a source of disagreement. E.g., in the quote below in which the father seems to take an overall perspective as concerns the infant’s transition whereas the mother focuses on the meal as such.

Mother: It upsets me that he doesn’t want my food and kind of annoys me that he doesn’t sit like an eager young bird, beak wide open, waiting to be fed like you see on films, and then I get worried because he doesn’t get anything to eat.

Father: The way I see it, there’s no need to worry. Well, he has enough supply for the winter, and we still have more than half the supply left.

Mother: Maybe that is why I feel somewhat pissed off, because I don’t feel you take me seriously … when you say “take it easy” and things like that! I really do feel that this is serious … A MAJOR problem. Food!

Parents’ idealized images also constituted a manuscript for how a “normal” meal should proceed. When parents found their infants ate sparsely they used their creativity to find ways in which they could cajole their infant into eating. Instead of spoon-feeding, some parents placed the food on the table in front of the infant. Others tried to divert the infant although this compromise on their idealized images for a “normal” meal. However, the infant’s food intake took priority and legitimized this deviation.

Mother: I wanted him to look me in the eye when I fed him, so he understood what we were doing, but we couldn’t get any food into him and then you try something else … As soon as he got something in his hands, he opened his mouth and took the food automatically and without problems, but then I feel I’m cheating him.

## Communicating meal-related values to the infant

The transition phase appeared as an opportunity to communicate to the infant good and healthy eating habits. Eating together with the infant appeared for parents as a significant part of becoming a real family and as an opportunity to familiarize the infant with different types of taste and texture to prevent picky eating.

Parents strived to communicate a pleasant sense of togetherness during meals and avoid negative experiences during meals. Especially if the parents themselves belonged to a family of picky eaters with negative childhood meal experiences.

Father: We have talked about your family being picky eaters, and you were a very picky eater yourself.

Mother: Yes, my mother insisted that I eat certain vegetables and eventually I had to revolt against her.

Positive meal -related values were communicated to the infant when the infant was happy and relaxed and willingly consumed the food served by the parents. In other words when the mood during the meal was pleasant. However, communicating meal-related values to the infant could at times be a challenge. The parents could find it difficult to teach the infant how to adopt an open mind towards food without pressuring the infant.

Father: Meals are of a very varying nature … whether they are pleasant and relaxed, or it’s just about getting some food into him, or to trick him into eating something.

Mother: The main thing is that eating doesn’t upset him … that he wants to eat and that he gets a varied diet and wants to try new things.

## Discussion

Our findings of parents’ lived experience of the transition from milk to solid food illustrated that during this transition, parents and infant alike had to find new ways of feeding that differed from breast or bottle feeding. The transition was complex, not only because it depended on the success of the parents’ endeavours, but also on the infant’s appetite and ability to cooperate.

When starting their infant on solid foods contrasting feelings aroused: sometimes the process was experienced as exciting, while at other times it connoted feelings of pressure at both personal and social levels. Research shows that parents often describe their infant as being fussy, temperamental and of varying appetite (McMeekin et al., 2013; van Dijk et al., 2009; Wasser et al., 2011). According to guidelines, parents are recommended to practice responsive feeding by, e.g., feeding their child slowly, encourage eating, maintaining eye contact and responding to the infant’s expressions (WHO, 2005, 2018). The recommendation of this responsive approach presupposes that parents are able to read the infant’s cues and respond appropriately. However, the findings in the present study showed that the recommended approach of responsive feeding was difficult for parents to follow as infants’ responses were difficult for parents to decode, due to the infants’ changing reactions to meals and the lack of a linear progression.

Including both parents’ experiences of the transition phase contributed with new knowledge of mutual parenting during the transition. We found that parents had idealized images about what constituted a “normal” meal. Parents’ idealized images were strongly influenced by personal perceptions and mirrored their view of progression in the transition period. Parents’ self-constructed images thus contributed to insecurity as well as feelings of pressure or disagreement when their images differed or deviated from their actual experiences. In connection with these findings, it was a paradox that parents who needed information often experienced that the information given made them insecure due to conflicting advice. Different or opposing types of information may underpin a feeling of uncertainty, especially when parents are trying to familiarize themselves with a new topic (Nielsen, Michaelsen, & Holm, 2013; Synnott et al., 2007). This may explain why some parents were left confused in our study. Thus, our findings suggest that health professionals need to be aware of parents’ unrecognized idealized images in order to broaden parents’ perception of what constitutes a normal meal.

Another main finding in the present study concerned that parents were alert and felt anxious when they introduced the infant to new food. Studies have shown that the parents’ behaviour is important for the infant’s acceptance of food (Bentley, Wasser, & Creed-Kanashiro, 2011; Mitchell, Farrow, Haycraft, & Meyer, 2013). It is likely that positive expressions as well as a positive tone of voice increase the infant’s acceptance of the eating situation. In the present study, the face of the parent likely mirrored their alertness and may therefore have signalled to the infant: “this is risky, you should be careful”; in other words: “do not eat”. The findings call for health professionals to increase the focus on guiding parents, allowing them to better read their infants’ cues and to respond adequately to them in the transition phase.

Including both parents’ experiences of the transition phase also afforded insight into their interaction and revealed both opposed and shared parental perspectives on the transition phase. Although the present study considered both parents’ experiences the findings revealed that the mothers, not only were the main responsible for teaching the infant to eat solid foods; she also had to provide varied and healthy food; otherwise she may be responsible for developing unhealthy eating habits according to recommendations and research (Schmid et al., 2011; Shim et al., 2011). In our study these recommendations were reflected differently among the parents. The mothers were particularly concerned with all of the details related to the infant’s food intake, whereas the father’s role tended to consist in backing up the mother in her choices and attempts to be a responsible and perfect mother. These findings may explain why research on parental roles has concluded that mothers call for support from health professionals in the transition phase (Bramhagen, Axelsson, & Hallström, 2006), whereas fathers want health professionals to involve them further in the transition phase (Mercer, 2004). Corresponding with these findings our findings further underline how a crucial part of the feeling of being a good parent consists of being able to feed the infant and to make it feel comfortable.

## Study limitations

In Reflective Lifeworld Research the scientific quality is evaluated by the criteria of objectivity, validity and generalization, however, the criteria are mediated to fit a phenomenological approach (Dahlberg et al., 2008; van Wijngaarden, van der Meide, & Dahlberg, 2017). The open and sensitive attitude adopted, the scrutinizing of our emerging understanding, and our constant critical reflection were important factors during the process of achieving objectivity and validity as they were helpful to distance ourselves and focus on how meanings come to be.

Nevertheless, the interviews were complicated by the fact that both parents were present during the interviews, meaning that different perspectives sometimes were at play simultaneously. Although joint interviewing is recommended when the phenomenon under study is a shared experience (Norlyk et al., 2016), and although both parents were present during the interviews, the interviews reflected that mothers were the more active respondents. Thus, they might have dominated the discussions. Accordingly, the findings unintentionally may reflect mothers’ perspectives over the fathers’.

In some cases, the interviewer (JSL) addressed the mother only. It cannot be determined from the data if this is an expression of habit, as health visitors usually communicate with the mother, or of a dominating woman-to-woman relationship or, potentially, a valid expression of the finding in this study of the father’s subordinate role, providing support for the mother. On the other hand, interviewing parents together contributed to generate data that would not be obtained through individual interviews.

The articulation of the essential structure lifts the findings above the concrete level of individual experiences. However, essential meanings as well as individual experiences are contextual (Dahlberg, 2019; Dahlberg et al., 2008). The present findings reflect a Danish context, which may limit the transferability as parents’ expectations and reactions to infants’ behaviour during the transition are likely to be culture-dependent (Synnott et al., 2007).

## Conclusion

The transition from milk to solid food was characterized as a demanding in-between phase that required parents’ full attention and sensitivity towards the infant’s food, intake and progress as well as the infant’s reactions to unfamiliar food. Consequently, the transition had a strong presence in the parents’ everyday life.

The findings contribute with new knowledge of the transition process by highlighting how shared parental experiences were reflected in frames for how a meal should normally proceed, including parents’ desire to create healthy eating habits and uphold harmony during family meals.

In brief, our findings suggest for health professionals to present parents with a wider frame of normality, especially as concerns the concept of what constitutes “normal” eating patterns.
